# Introduction to the special issue from the proceedings of the 2006 International Workshop on Virtual Reality in Rehabilitation

**DOI:** 10.1186/1743-0003-4-18

**Published:** 2007-06-06

**Authors:** Emily A Keshner, Patrice (Tamar) Weiss

**Affiliations:** 1 Dept. of Physical Therapy, College of Health Professions, Temple University, Jones Hall 600, 3307 Broad St., Philadelphia, PA 19140, USA; 2 Dept. of Electrical and Computer Engineering, College of Engineering, Temple University, 1947 North 12th St., Philadelphia, PA 19122, USA; 3 Laboratory for Innovations in Rehabilitation Research, Dept. of Occupational Therapy, Faculty of Social Welfare & Health Sciences, University of Haifa, Mount Carmel, Haifa 31905, Israel

## Abstract

New technologies are rapidly having a great impact on the development of novel rehabilitation interventions. One of the more popular of these technological advances is virtual reality. The wide range of applications of this technology, from immersive environments to tele-rehabilitation equipment and care, lends versatility to its use as a rehabilitation intervention. But increasing access to this technology requires that we further our understanding about its impact on a performer. The International Workshop on Virtual Reality in Rehabilitation (IWVR), now known as Virtual Rehabilitation 2007, is a conference that emerged from the need to discover how virtual reality could be applied to rehabilitation practice. Individuals from multiple disciplines concerned with the development, transmission, and evaluation of virtual reality as a technology applied to rehabilitation attend this meeting to share their work. In this special issue of the Journal of NeuroEngineering and Rehabilitation we are sharing some of the papers presented at the 2006 meeting of IWVR with the objective of offering a description of the state of the art in this research field. A perusal of these papers will provide a good cross-section of the emerging work in this area as well as inform the reader about new findings relevant to research and practice in rehabilitation.

## Background

The most influential change in physical rehabilitation practice over the past decade has been the vast development of new technologies that enable clinicians to provide a greater variety of therapeutic interventions. Virtual reality is fast becoming one of the more popular of these new technologies. Reasons for its popularity include its potential for providing safe and functional environments that can be adapted to meet the needs of diverse therapeutic objectives.

The International Workshop on Virtual Reality in Rehabilitation (IWVR) is a conference that emerged from the need for the disciplines concerned with the development, transmission, and evaluation of virtual reality as a technology applied to physical rehabilitation to have an opportunity to educate and learn from each other. Improvements in technology depend on interdisciplinary cooperation among neuroscientists, engineers, computer programmers, psychologists, and rehabilitation specialists, and on adoption and widespread application of objective criteria for evaluating alternative methods [1]. If technology transfer is to become successful, we need to establish collaborative interactions in which the engineers, clinical scientists, and clinicians are comfortable with the language of each respective discipline, and in which the goals for each discipline becomes overlapping with the skills and goals of the other fields of endeavor and of the consumer [2]. Such interdisciplinary interactions are demonstrated in the work presented in most of the papers contained in this special issue of the Journal of NeuroEngineering and Rehabilitation. One glance at the academic affiliations of the authors of these papers will reveal how important multidisciplinary input is in order to make these new technologies relevant to the problems encountered in rehabilitation.

## Virtual reality as a treatment intervention

The four full papers and five short communications included in this issue span a wide range of clinical areas that can be impacted by virtual reality. Several of these papers have demonstrated that a virtual reality intervention can produce a significant clinical impact. Bugnariu and Fung describe the results of a study on the effects of unanticipated visual feedback on aging, and the capability of the central nervous system to select pertinent sensory information after repeated exposure to sensory conflicts created by VR.

Lamontagne, Fung, McFadyen and Faubert used a virtual environment to investigate how perception of optic flow speed influenced the walking speed of patients who have had a stroke. Stewart, Yeh, Jung, Yoon, Whitford, Chen, Li, McLaughlin, Rizzo, and Winstein describe four novel VR tasks that were developed and tested to improve arm and hand movement skills in individuals with hemiparesis. Subramanian, Knaut, Beaudoin, McFadyen, Feldman and Levin describe an immersive and interactive experimental protocol developed in a virtual reality environment and designed to provide feedback about performance and quality of pointing movements to patients with motor disorders.

## Virtual reality as a evaluative tool

Virtual reality has also been explored as a tool to assess disability. Keshner, Streepey, Dhaher, and Hain used a virtual environment to measure the combined effect of visual and physical disturbances on postural response kinematics, and found that responses of individuals with visual sensitivity could be distinguished by their diagnostic histories. Morganti, Gaggioli, Strambi, Rusconi and Riva present an integrated evaluation method in which neuropsychological spatial ability evaluation is extended with more situational computer based tools that allow the assessment of spatial orientation during the interaction with complex 3D environments.

## Future directions

Another paper has focused on further developing the technology to make it more versatile. Oddsson, Karlsson, Konrad, Ince, Williams, and Zemkova present the design of a new portable device that was an extension of their research on making the protocol of partial body weight support during treadmill walking more functional by adding a virtual environment that stimulated associated postural adjustments.

Finally, two papers present very preliminary data that hint at the tremendous potential of this versatile technology for new areas of rehabilitation research. Baheux, Yoshizawa, and Yoshida have designed a virtual environment to simulate hemispatial neglect with the aim of providing a more precise diagnostic tool. Dvorkin, Kenyon, and Keshner shifted the orientation of both the central and peripheral visual fields in a virtual visual environment to determine how changes in visuo-spatial relations alter motor planning during reaching.

## Conclusion

This collection of papers provides an up-to-date description of the state of the art in the field of virtual reality as applied to rehabilitation. The field is rapidly advancing and numerous research groups have already demonstrated applications of great clinical relevance. It is our hope that the papers in this issue will not only educate the readers about the current state of research with virtual reality, but also generate new ideas that promote rehabilitation research and application using new technologies like virtual reality.
